# Evaluation of pre-Games effects of the Tokyo 2020 Olympic Games on Japanese population-level physical activity: a time-series analysis

**DOI:** 10.1186/s12966-022-01332-x

**Published:** 2022-08-06

**Authors:** Shiho Amagasa, Masamitsu Kamada, Adrian E. Bauman, Motohiko Miyachi, Shigeru Inoue

**Affiliations:** 1 Graduate School of Public Health, Teikyo University, 2-11-1 Kaga, Itabashi-ku, Tokyo, 173-8605 Japan; 2 Department of Health Education and Health Sociology, School of Public Health, Graduate School of Medicine, The University of Tokyo, 7-3-1 Hongo, Bunkyo-ku, Tokyo, 113-0033 Japan; 3 Department of Preventive Medicine and Public Health, Tokyo Medical University, 6-1-1 Shinjuku, Shinjuku-ku, Tokyo, 160-8402 Japan; 4 Prevention Research Collaboration, School of Public Health, University of Sydney, Sydney, NSW 2006 Australia; 5 Faculty of Sport Sciences, Waseda University, 2-579-15 Mikajima, Tokorozawa, Saitama 359-1192 Japan

**Keywords:** Olympic Legacy, Time-series Analysis, Epidemiology, Exercise, Health Promotion

## Abstract

**Background:**

The Olympic Games represent an opportunity to create a ‘physical activity legacy’ that promotes physical activity at the population level in the host nations and cities. However, previous studies showed little increase in population-level physical activity following the Olympics. The upsurge of public interest in sports and physical activity participation before the Olympics may diminish rapidly following the Games. We examined the pre-Games effects of the Olympics on Japanese population-level physical activity after the announcement of Tokyo’s successful bid for the 2020 Olympic and Paralympic Games in September 2013.

**Methods:**

We used publicly available data from serial cross-sectional surveys conducted with nationally or regionally representative samples in Japan seven years before and after the announcement (from 2006–2020). The outcomes were 1) daily step counts and 2) exercise habit prevalence (≥ 30 min/day, ≥ 2 days/week, and over a year) from the National Health and Nutrition Surveys Japan (NHNS-J; 14 time points; aggregated data); and 3) sports participation (at least once a week) from the National Sports-Life Survey conducted every two years (NSLS; eight time points; individual-level data of 18,867 adults) and from the Public Opinion Survey on Sports Participation of Tokyo Residents (POSSP; eight time points; aggregated data). Age- and gender-adjusted regression models were used to estimate changes in the outcomes before and after the announcement.

**Results:**

There were no significant pre-Games effects of the Olympics on national-level physical activity participation among Japanese adults. Sports participation (56.4% and 57.5%, respectively; *P* = 0.518), daily steps (6,535 and 6,686 steps/day; *P* = 0.353), and exercise habit (30.7% and 29.1%, *P* = 0.309) did not change significantly before and after the announcement. Although an increase in sports participation among Tokyo residents was not found in the NSLS (61.5% and 59.3%, *P* = 0.227), it was observed in the POSSP (49.1% and 57.7%, *P* = 0.019). Nonetheless, this increase might not be related to the pre-Games effects since the trend diminished following the announcement.

**Conclusions:**

Population-level physical activity did not show significant changes until 2020. Realising the physical activity legacy of an Olympics may require strategic promotion and cross-agency partnership implementation in the pre- and post-event period.

**Supplementary Information:**

The online version contains supplementary material available at 10.1186/s12966-022-01332-x.

## Introduction

Hosting mega sporting events, such as the Olympic and Paralympic Games, can provide host nations and cities with opportunities to create health and physical activity legacies that yield long-term benefits. Recently, the Olympic Bid and planning documents from host cities have frequently mentioned population-level targets for physical activity and/or sports participation as planned legacies of the event [1, 2]. However, previous studies have shown that, since 1996, changes in population-level physical activity participation immediately before or after the Olympic Games have been minor [1, 3, 4]. In particular, two studies conducted with representative adult or children samples evaluated the impact of the Sydney 2000 Olympic Games [4] and Vancouver 2010 Olympic Games [3] on physical activity after the events. Neither resulted in improved physical activity participation. Further, although some studies showed a temporary increase in people’s interest and intention to be active before and after the Games, these effects did not lead to behavioural changes (i.e. physical activity participation) at the population level [1, 4]. Research suggests that these mega sporting events need to be strategically complemented with planned approaches that might stimulate policy action several years before the Games to encourage sports and physical activity participation among the population [5].

In September 2013, Tokyo won the bid to host the 2020 Olympic and Paralympic Games. A key concept for the bid application included recovery and reconstruction from the 2011 Great East Japan Earthquake [6]. After the announcement, the momentum for sports and sports participation promotion increased in Japan. In addition, based on the principles of the Basic Act on Sport enacted in 2011, Japan aimed to realize societal health and longevity through sports, which can play an important role in maintaining and improving mental and physical health [7]. In October 2015, the Japan Sports Agency (JSA) was established as an external bureau of the Ministry of Education, Culture, Sports, Science and Technology to comprehensively promote sports and sports participation towards the 2020 Games and after by leading a collaboration between the relevant government agencies [8]. In 2016, the Tokyo Organizing Committee of the Olympic and Paralympic Games set up the ‘Action & Legacy Plan’ to create legacies for the 2020 Games. The Plan aimed to take comprehensive action around five themes, such as ‘Sport and Health’, which encompassed interventions to increase sports participation by creating a society where everyone could play, watch, and support sports [9]. For example, sports events and programs were implemented in schools and communities prior to the Games [9].

Within these contexts, previous studies mainly focused on the comparison of physical activity or sports participation before and after the Olympic Games [1, 3, 4, 10, 11]. The upsurge of public interest in sports and physical activity participation may occur before the Games, and then gradually diminish after. Additionally, some promotional programs were conducted prior to the Games [9]. Notwithstanding, the ‘pre-Games effect,’ i.e., the effect following the announcement of the host city and preceding the Olympic Games, on population-level physical activity participation has not been examined. Therefore, using secondary national and local surveillance data, we aimed to evaluate the pre-Games effects of the Tokyo 2020 Olympic Games on population-level physical activity and sports participation after the announcement of the successful bid in September 2013.

## Methods

### Population-level physical activity data

We used publicly available physical activity data from serial cross-sectional surveys conducted with nationally or regionally representative samples in Japan before and after the announcement of Tokyo’s successful bid for the 2020 Olympic and Paralympic Games in September 2013. Physical activity outcomes included sports participation, daily step counts, and exercise habit prevalence.

Specifically, we used data from the National Health and Nutrition Survey Japan (NHNS-J) 2006–2019 (14 time points, aggregate data), National Sports-Life Survey (NSLS) 2006–2020 (eight time points, both aggregate- and individual-level data), and Public Opinion Survey on Sports Participation of Tokyo Residents (POSSP) 2009–2018 (six time points, aggregate data). Since consistency was critical to evaluate long-term trends in survey-based methodologies, we only included surveys with no or minor changes in methods and excluded those with major changes (e.g. surveys by the JSA, which changed the definition of sports in 2017).

#### NHNS-J

The NHNS-J was a cross-sectional interview and examination survey conducted annually in November by the Ministry of Health, Labour and Welfare (MHLW). Its methodological details have been described in previous research [12, 13]. Briefly, in the NHNS-J, stratified random sampling was used to obtain a nationally representative sample. Each year, 300 of approximately 5,000 census units were randomly selected based on the census unit targeted in the Comprehensive Survey of Living Conditions. In 2012 and 2016, the NHNS-J surveys had larger sample sizes to enable the comparison of the results across all 47 Japanese prefectures. Following the Great East Japan Earthquake and Kumamoto Earthquake, typhoon, and Tottori Earthquake, the affected areas were excluded from the 2011/2012 surveys and 2016, respectively.

The NHNS-J has been used to investigate adult exercise habit prevalence and daily steps since 1986 and 1989, respectively. Based on the protocol set by the MHLW, regional health centres with authority over the area provided venues to conduct the survey. The 2020 and 2021 NHNS-J were cancelled due to the COVID-19 pandemic.

Exercise habit was defined as doing exercise for ≥ 30 min/day, ≥ 2 days/week, over a year. This variable was assessed by face-to-face structured interviews. Participants were asked to visit a designated facility within walking distance of their residence on a scheduled date. Until the 2012 NHNS-J, participants chose the answer that most accurately described their exercise habits: 1) unable to exercise due to a health problem, 2) unable to exercise due to other problems, and 3) exercised for ≥ 30 min/day, ≥ 2 days/week, over the previous year. Since 2013, participants reported their exercise frequency (days/week), duration (minutes/day), and period (years) if exercise was not contraindicated by their doctor. The aggregate exercise habit data, which were comparable to those before 2012, were calculated by the MHLW. We used the prevalence data (aggregate data) on exercise habits from the annual NHNS-J reports.

Daily step counts were measured using an AS-200 pedometer (Yamasa Co. Ltd., Tokyo, Japan) throughout a single day for each participant. The pedometers of Yamasa Co. Ltd. are widely used in research and internationally known as Yamax [14]. The pedometer surveys were conducted annually on a single day between Monday and Saturday in November, and participants selected a single typical day during this period to be monitored in their physical activity using the pedometer. They wore the device on their waist while awake, except for any time spent in water-based activities, and then recorded their steps on a survey log. Since 2012, participants were restricted to adults aged ≥ 20 years and data from participants who took < 100 or ≥ 50,000 steps/day were excluded from the national estimate calculation. We used the average value of daily steps from the annual NHNS-J reports.

#### NSLS

The NSLS has been conducted by the Sasakawa Sports Foundation every two years since 1992 [15, 16]. It included various items focused on sports participation. In the NSLS from 2008–2020, data from 2,000 or 3,000 participants (≥ 20 years) were collected by quota sampling and placement method (leaving method) annually. Particularly, the investigator visited the respondents and asked them to complete the paper-based questionnaire within a certain time interval [15, 16]. In the 2006 NSLS, 3,000 adults aged ≥ 20 years were selected by two-stage stratified random sampling, and 1,867 responded to the survey. In the questionnaires, participants were asked to report all the sports in which they participated during the previous year from a list, as well as on the frequency and duration of their participation in each sport [16]. The 2020 NSLS was conducted between late August and mid-September, when COVID-19 infection rates were relatively low (3.4–6.3 new confirmed cases per million) [17] and Japan was not under a national state of emergency (see below for sensitivity analyses). We used public, de-identified, individual-level data on sports participation and sociodemographic characteristics (i.e. age, gender, and residential area) available from the Sasakawa Sports Foundation’s website [18].

#### POSSP

The POSSP has been conducted by the Tokyo Metropolitan Government approximately every two years since 2007. Using two-stage stratified random sampling, 3,000 adults aged ≥ 20 years (or ≥ 18 years) in Tokyo were selected for the survey. Next, face-to-face interviews were conducted to assess sports participation, where respondents chose the sports and exercises in which they participated in the past year from a list of sports and exercise types. Those who reported they participated in sports and exercises were then asked to choose the option that most accurately represented their activity frequency: ≥ 3 days/week, 2 days/week, 1 day/week, 1–3 days/month, 1–2 days per 3 months, 1–3 days per year. We used the prevalence data (aggregate data) on sports participation from the POSSP reports.

### Statistical analysis

Data on population-level physical activity participation and sociodemographic characteristics were obtained from the report of each survey for each year. We described the number of participants, physical activity participation data, proportion of males, and mean age or proportion of older adults (60 years and older) in each survey. Age- and gender-adjusted regression models were used to estimate changes in physical activity outcomes before and after the announcement of the successful bid. The NSLS data were adjusted for residential areas. The mean absolute change and 95% confidence intervals were calculated using linear regression.

Multilevel analysis was applied for individual-level data from the NSLS, using the survey year as a random effect. A segmented regression approach was also performed to test the change in slope (‘segmented’, R Package), used to identify underlying trends during the analysed period (i.e. from before to after the announcement of the successful bid) [19, 20]. Analyses were performed using SAS, version 9.4, (SAS Institute Inc., Cary, NC) and R, version 3.5.2, (R Foundation for Statistical Computing, Vienna, Austria). The significance level was set at 5%.

Since the NSLS changed its sampling methods (i.e. two-stage stratified random sampling in 2006 and quota sampling from 2008 onwards), we conducted a sensitivity analysis using data from 2008 to 2020 (seven time points). We also conducted another sensitivity analysis that excluded the data for 2020, as the COVID-19 pandemic may have affected sports participation during the year (i.e. 2008–2018; six time points).

## Results

The characteristics of the survey data are presented in Table 1. The number of participants, proportions by gender, and age distribution differed by survey. Data excluded from the years in which the NHNS-J was in its expanded format (i.e., 2012 and 2016), the average number of participants in the pedometer and adult exercise habit surveys were 6,405 (standard deviation [SD] = 967) and 4,176 (SD = 711), respectively. There were slightly more female than male participants, and the proportion of people aged 60 years and older increased over the years. In the POSSP and NSLS, the average number of participants were 1,909 (SD = 96) and 2,358 (SD = 533), respectively.

**Table 1:** Demographic characteristics of the respondents of all the surveys included in this study by year and outcome measure

	NHNS-J						NSLS							POSSP		
	Step count (nationwide)			Exercise habit (nationwide)			Sports participation (nationwide)				Sports participation (Tokyo)			Sports participation (Tokyo)		
Year	n	% Male	% Older adults	n	% Male	% Older adults	n	% Male	Mean age (SD)	% Urban residents	n	% Male	Mean age (SD)	n	% Male	Mean age
2006	7,522	46.0	36.9	4,968	41.6	44.6	1,867	47.9	51.0 (17.2)	23.2	161	45.3	50.6 (17.2)			
2007	7,131	45.7	38.6	4,817	41.9	46.6										
2008	7,459	45.4	43.4	5,057	42.0	52.5	2,000	49.4	48.9 (16.5)	25.0	202	50.5	47.1 (16.6)			
2009	7,320	45.4	41.2	4871	41.3	49.6								2,079	45.8	34.3
2010	7,141	46.1	42.1	4,621	42.5	51.2	2,000	49.2	49.3 (16.7)	26.2	224	50.9	46.9 (16.4)			
2011	6,712	45.5	42.1	4,188	42.2	52.5								1,896	49.5	39.0
2012	24,369^a^	45.8	46.9	16,595^a^	41.8	53.2	2,000	49.5	49.5 (16.7)	28.1	209	52.2	48.8 (17.5)	1,928	51.4	41
2013	6,084	46.2	48.6	3,864	43.1	55.2										
2014	6,330	46.4	49.9	4,100	43.4	55.9	2,000	49.5	50.1 (16.7)	28.5	219	50.7	47.8 (16.5)	1,910	50.4	40.1
2015	5,858	45.8	47.9	3,879	41.8	55.0										
2016	20,236^a^	45.7	50.0	13,604^a^	42.4	57.2	3,000	49.7	49.3 (17.0)	28.7	310	50.6	47.4 (16.6)	1,820	47.7	36.3
2017	5,380	46.5	49.7	3,464	43	56.4										
2018	5,356	46.7	48.1	3,466	42.3	55.6	3,000	49.7	49.4 (16.8)	29.3	330	51.2	45.8 (15.9)	1,818	49.4	38.4
2019	4,568	46.5	51.4	2,814	43.3	59.6										
2020							3,000	49.8	49.8 (16.9)	29.6	320	50	47.0 (16.9)			
Mean (SD)	6405 (967)^b^	46.0 (0.4)	45.5 (4.7)	4176 (711)^b^	42.3 (0.7)	53.2 (4.1)	2358 (533)	49.3 (0.6)	49.6 (0.6)	27.3 (1.7)	247 (64)	47.7 (1.5)	47.3 (1.0)	1,909 (96)	49.0 (2.0)	38.2 (2.5)

*NHNS-J* National Health and Nutrition Survey, Japan, *NSLS* National Sports-Life Survey, *POSSP* Public Opinion Survey on Sports Participation of Tokyo residents, *SD* Standard deviation

^a ^Year in which the survey was in its expanded format

^b ^Data from the years in which the survey was in its expanded format were excluded. The proportion of older adults (aged ≥ 60 years) was calculated based on the data from those who participated in each survey

**Fig.1:**
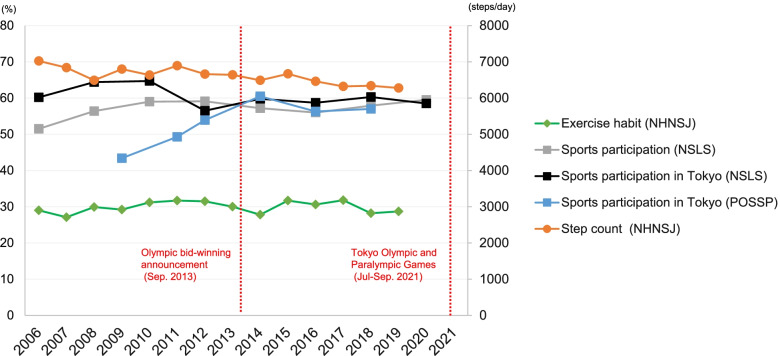
Population-level physical activity before and after the announcement of Tokyo’s successful bid for the 2020 Olympic Games in Japan. NSLS, National Sports Life Survey; NHNS-J, National Health and Nutrition Survey, Japan; POSSP, Public Opinion Survey on Sports Participation of Tokyo residents. Sports participation rate is defined as engaging in sports at least once per week. Exercise habit prevalence was defined as ≥ 30 min/day, ≥ 2 days/week, over a year. The prevalence and average step data were not adjusted

Figure 1 describes the trajectory of the average step count, exercise habit prevalence, and sports participation rate between 2006 and 2020. There were no significant pre-Games effects of the Olympic Games on population-level physical activity outcomes among adults. Sports participation did not change significantly between 2006–2012 and 2014–2020 (56.4% and 57.5%, *P* = 0.518), nor did daily steps (6,535 and 6,686 step/day, *P* = 0.353) or exercise habits (30.7% and 29.1%, P = 0.309) between 2006–2012 and 2013–2019 (Table 2) before and after the announcement.

**Table 2:** The pre-Games effects of the Tokyo 2020 Olympic Games on Japanese population-level physical activity participation

			**Pre (2006–2012)**		**Post (2013–2020)**		**Change**	
	**Survey year**	**Data**	**Mean**^a^	**(95% CI)**	**Mean**^a^	**(95% CI)**	**Mean**	**(95% CI)**
**NHNS-J**								
Step count (steps/day)								
Overall	2006–2019	A	6,535	(6,395; 6,676)	6,686	(6,546; 6,827)	151	(-195; 497)
Male	2006–2019	A	7,116	(6,986; 7,247)	7,108	(6,977; 7,239)	-9	(-304; 287)
Female	2006–2019	A	6,182	(6,012; 6,352)	6,188	(6,018; 6,357)	6	(-376; 387)
Exercise habit prevalence (%)								
Overall	2006–2019	A	30.7	(29.3; 32.0)	29.1	(27.8; 30.5)	-1.5	(-4.7; 1.7)
Male	2006–2019	A	34.8	(32.9; 36.6)	32.3	(30.5; 34.2)	-2.4	(-6.5; 1.6)
Female	2006–2019	A	28.0	(26.9; 29.2)	26.3	(25.2; 27.5)	-1.7	(-4.2; 0.7)
**NSLS**								
Sports participation (%)								
Overall	2006–2020	A	58.6	(57.0; 60.2)	55.6	(54.0; 57.2)	-3.0	(-8.7; 2.6)
Male	2006–2020	A	56.0	(54.8; 57.2)	56.6	(55.4; 57.9)	0.6	(-2.2; 3.5)
Female	2006–2020	A	56.8	(54.2; 59.4)	58.8	(56.2; 61.4)	2.0	(-4.0; 8.1)
Sports participation (%)								
Overall	2006–2020	I	56.4	(55.3; 57.6)	57.5	(56.4; 58.5)	1.1	(-2.1; 4.3)
Male	2006–2020	I	55.9	(54.2; 57.6)	56.5	(55.1; 58.0)	0.7	(-1.7; 3.1)
Female	2006–2020	I	57.0	(55.4; 58.7)	58.4	(56.9; 59.8)	1.4	(-2.6; 5.4)
Sports participation (%) in Tokyo (sub-sample)								
Overall	2006–2020	I	61.5	(58.1; 64.9)	59.3	(56.5; 62.1)	-2.2	(-5.7; 1.4)
Male	2006–2020	I	61.3	(56.4; 66.1)	54.9	(50.9; 58.9)	**-6.4**	(**-11.0; -1.8)**
Female	2006–2020	I	61.8	(57.0; 66.5)	63.8	(59.9; 67.7)	2.0	(-2.9; 6.9)
**POSSP**								
Sports participation (%) in Tokyo								
Overall	2009–2018	A	49.1	(48.2; 50.0)	57.7	(56.8; 58.6)	**8.6**	(**3.4; 13.8)**
Male	2009–2018	A	47.2	(45.2; 49.1)	54.4	(52.5; 56.4)	**7.3**	(**0.7; 13.9)**
Female	2009–2018	A	49.7	(47.9; 51.4)	62.2	(60.5; 64.0)	**12.6**	(**6.4; 18.8)**

*NHNS-J* National Health and Nutrition Survey Japan, *NSLS* National Sports-Life Survey, *POSSP* Public Opinion Survey on Sports Participation of Tokyo residents, *CI* Confidence interval, *A* Aggregated data, *I* Individual data

^a ^Mean values and regression models were adjusted for age and gender. We also adjusted for residential areas when analysing the NSLS data

Bold indicates *P* < 0.05

The sub-sample analysis of the NSLS (between 2006–2020) showed a slight decrease in sports participation rates among male adults living in Tokyo (61.3% and 54.9%, *P* = 0.007), while no significant changes were found among their female counterparts (61.8% and 63.8%, *P* = 0.419). In the POSSP, there was an increase in sports participation in Tokyo (49.1% between 2009–2012 and 57.7% between 2014–2018, *P* = 0.019), although this increase might not be attributable to pre-Games effects as it preceded the announcement of the successful bid (estimated break-point = October 2013, *P *= 0.037, Additional file 1: Appendix Fig. 1).

In the sensitivity analysis using the NSLS data, similar findings were observed, except for sports participation among male adults in Tokyo (Additional file 2: Appendix Table 1). When data were restricted to the period of 2008–2018, we no longer observed a significant difference in sports participation rates among male adults in Tokyo. However, we observed a significant decrease in national-level sports participation rate among male adults (57.2% and 56.1%, *P* = 0.006) before and after the announcement.

## Discussion

Using data collected from Japanese adults several years before and after the announcement of Tokyo’s successful bid for the 2020 Olympic and Paralympic Games in 2013, we observed no significant pre-Games effects of the Games on national- and local-level physical activity participation. To the best of our knowledge, this is the first study to evaluate the pre-Games effects of the Olympics on population-level physical activity participation.

While looking ahead to the Tokyo 2020 Games, the Action & Legacy Plan proposed a range of initiatives to promote physical activity in the Japanese population [9]. However, limited processes were established to assess the extent to which these ‘actions’ were implemented, their effectiveness in reaching the entire Japanese population, or whether the legacy was created [9]. While the Tokyo 2020 Action & Legacy Report stated that one of the key achievements was “the ratio of people who conduct sports, which was 42.5% in 2016, became 59.9% in 2020” [21], sport participation rate in the JSA survey was not appropriate for legacy assessment since the definition of sport and methodology was changed during the survey, as noted in the methods. If most of these Olympics-related initiatives were one-time events and not accompanied by strategic and integrated approaches that targeted those who were physically inactive, the physical activity legacy anticipated by the Action & Legacy Plan may not be realised. The Organizing Committee for the Games, International Olympic Committee, the host country's Olympic Committee, national and regional administrative agencies, and sponsor companies should work together strategically so that well-designed social marketing campaigns and comprehensive and continued community-based programs can leverage the Olympic momentum and increase population-level physical activity participation. To assess these effects, a comprehensive process and impact evaluation framework should be implemented throughout the pre- and post-event period [1, 4, 5, 22].

Regarding sports participation among Tokyo residents, the results were not exactly concordant among the surveys. This may owe to methodological differences between the NSLS and POSSP, which included in sampling (quota sampling vs. two-stage stratified random sampling), techniques (placement method vs. interview technique), items on sports participation (i.e. with different formats), and participants’ sociodemographic characteristics. Importantly, the sub-sample analysis of the NSLS had a smaller sample size, and the sensitivity analyses showed results that were less stable.

Prior to 2020, Japan had been the host country for three other Olympic Games: the Tokyo 1964 Summer Olympics, the Sapporo 1972 Winter Olympics, and the Nagano 1998 Winter Olympics. All such events were accompanied by various policies and initiatives related to physical activity and sports in response to the hosting of the Olympics [23, 24]. In particular, the Tokyo 1964 Summer Olympics had a substantial effect on the physical activity policy and surveillance systems in Japan (e.g. the Sports Promotion Act enacted in 1961). A previous study showed that individuals who experienced this 1964 event in their youth participated in sports more frequently than those from other generations, described as a long-term cohort effect [25]. The Games might leave a positive impression of sports in the minds of the young people, which may influence their subsequent attitudes toward sports, and sports participation [25]. Nonetheless, our study revealed a lack of clear positive pre-Games effects regarding the Tokyo 2020 Olympic Games. Hence, the evidence presented in this paper suggests the need for a long-term evaluation and ongoing surveillance of the impact of the Tokyo 2020 Olympic Games on population-level physical activity participation in Japan and Tokyo.

### Strengths and limitations

A major strength of this study is that the physical activity surveillance system in Japan allowed us to assess long-term trends in physical activity using different surveys (i.e. sports participation, exercise habit prevalence, and daily step). In the NHNS-J, the same pedometer has been used for more than 30 years, which provided a consistent device-based data set not easily nor usually found [26]. Although it was not possible to set up a control group, the existence of these long-lasting national surveys is essential to monitor population responses to mega sporting events, such as the Olympic Games.

However, some limitations of this study should be noted. First, we mainly used aggregate data. Hence, although our research can be compared to prior research that examined the impact of the Olympic Games on physical activity [1], more precise and detailed analyses data may be required, such as studies using individual-level data sets, for us to grasp this impact more comprehensively. Second, it was unclear how minor changes in assessment methodology in the NHNS-J (exercise habits in 2013; steps in 2012) might have affected the results. Still, the trends we observed did not show any apparent discrepancy before and after these changes. Third, we were not able to investigate the potential moderators of pre-Games effects, such as socioeconomic status. In the NSLS, from which we extracted individual-level data, there were data on educational attainment only from surveys conducted before 1996 or after 2014. Among these available data sets from the NSLS, we observed that inequality in sports participation by educational attainment remained evident, although sports participation rates had increased significantly by 2020 compared with 1996 (< 12 years of education: 37.5% in 1996 vs. 56.3% in 2020; ≥ 12 years: 46.7% vs. 62.7%). Future studies that include more sociodemographic measures may yield relevant data for assessing the impact of mega sporting events on physical activity inequality. Fourth, we acknowledge that natural disasters between 2006–2020 could have affected population physical activity as exogeneous events. However, no notable changes in national-level physical activity were observed before and after the years in which the NHNS-J sampling was affected by disasters. Finally, we did not examine the pre-Games effects of the Tokyo 2020 Paralympic Games on population-level sports participation among people with disability owing to the lack of data prior to the announcement of Tokyo’s successful bid. The Sport Basic Plan (the second term) by the JSA aimed to increase the proportion of people with disabilities who played sports at least once a week to 45.0% (25.3% in 2019) by March 2022 [8]. Thus, future research should assess how the hosting of the Tokyo 2020 Paralympic Games affected sports participation for people with disabilities.

## Conclusions

There were no significant pre-Games effects of the Tokyo 2020 Olympic Games on national- and local-level physical activity participation in Japan. Realising the physical activity legacy of an Olympic Games event may require more comprehensive strategic promotion and a sustained cross-agency partnership implementation throughout the pre- and post-Games periods.

## Supplementary Information

**Additional file 1: Appendix Figure 1.** Changes in the trends of population-level physical activity for each survey. NSLS, National Sports Life Survey; NHNS-J, National Health and Nutrition Survey, Japan; POSSP, Public Opinion Survey on Sports Participation of Tokyo residents; EBP, Estimated break-point. Sports participation rate is defined as engaging in sports at least once per week. For assessing exercise habit prevalence, exercise was defined as engaging in exercise activities for ≥30 min/day, ≥2 days/week, over a year. The exercise habit prevalence and average step count data were adjusted for age and gender. When analysing the NSLS data, the residential area was added to the adjustments. The estimated break-points and slope are calculated using segmented regression analysis. a)–c): national sample, d): Tokyo residents. For sports participation rates among Tokyo residents in the NSLS (sub-sample analysis, not shown), there was no estimated break point because there was no change in the linear relationship.

**Additional file 2: Appendix Table 1.** Sensitivity analysis for the pre-Games effects of the Tokyo 2020 Olympic Games on sports participation in the National Sports-Life Survey data set.

## Data Availability

The aggregated datasets used and analysed during this study are available from the corresponding author on reasonable request. Individual-level data of the NSLS are available upon the completion of the application on the Sasakawa Sports Foundation’s website (https://www.ssf.or.jp/thinktank/sports_life/application/index.html).

## Abbreviations

NHNS-J: National Health and Nutrition Survey, Japan
NSLS: National Sports-Life Survey
POSSP: Public Opinion Survey on Sports Participation of Tokyo Residents
SD: Standard deviation
JSA: Japan Sports Agency
MHLW: Ministry of Health, Labour and Welfare

## References

[1] Bauman AE, Kamada M, Reis RS, Troiano RP, Ding D, Milton K (2021). An evidence-based assessment of the impact of the Olympic Games on population levels of physical activity. Lancet.

[2] Ainsworth BE, Sallis JF. The Beijing 2022 Winter Olympics: an opportunity to promote physical activity and winter sports in Chinese youth. J Sport Health Sci*.* 2021. 10.1016/j.jshs.2021.09.005. 10.1016/j.jshs.2021.09.005 PMC8847916 34547481

[3] Craig CL, Bauman AE (2014). The impact of the Vancouver Winter Olympics on population level physical activity and sport participation among Canadian children and adolescents: Population based study. Int J Behav Nutr Phys Act.

[4] Bauman A, Bellew B, Craig CL (2015). Did the 2000 Sydney Olympics increase physical activity among adult Australians?. Br J Sports Med.

[5] Bauman A, Kamada M (2015). The potential effects of the Tokyo 2020 Olympic and Paralympic Games on physical activity participation at the population Level. Res Exer Epidemiol.

[6] The Tokyo Organising Committee of the Olympic and Paralympic Games. Tokyo 2020 Applicant City : application file by the Tokyo 2020 Bid Committee for the Games of the XXXII Olympiad and the XVI Paralympic Summer Games. https://library.olympics.com/Default/doc/SYRACUSE/62954/tokyo-2020-applicant-city-dossier-de-la-ville-requerante-du-comite-de-candidature-de-tokyo-2020-pour?_lg=en-GB. Accessed 8June 2022.

[7] Japan Sports Agency. Comprehensive promotion of sports administration in collaboration with relevant ministries. https://www.mext.go.jp/sports/en/about_us/collaboration/index.htm. Accessed 8 June 2022.

[8] Japan Sports Agency. https://www.mext.go.jp/sports/en/index.htm. Accessed 8 June 2022.

[9] The Tokyo Organising Committee of the Olympic and Paralympic Games. Tokyo 2020 Action and Legacy Plan 2016 : Participating in the Tokyo 2020 Games, connecting with tomorrow / The Tokyo Organising Committee of the Olympic and Paralympic Games https://library.olympics.com/Default/doc/SYRACUSE/166614/tokyo-2020-action-and-legacy-plan-2016-participating-in-the-tokyo-2020-games-connecting-with-tomorro. Accessed 8 June 2022.

[10] Weed M, Coren E, Fiore J, Wellard I, Chatziefstathiou D, Mansfield L (2015). The Olympic Games and raising sport participation: a systematic review of evidence and an interrogation of policy for a demonstration effect. Eur Sport Manag Q.

[11] Annear M, Sato S, Kidokoro T, Shimizu Y. Can international sports mega events be considered physical activity interventions? A systematic review and quality assessment of large-scale population studies. Sport Soc*.* 2021:1–18. 10.1080/17430437.2021.1957834

[12] Ikeda N, Takimoto H, Imai S, Miyachi M, Nishi N (2015). Data resource profile: The Japan National Health and Nutrition Survey (NHNS). Int J Epidemiol.

[13] Takamiya T, Inoue S (2019). Trends in step-determined physical activity among Japanese adults from 1995 to 2016. Med Sci Sports Exerc.

[14] Schneider PL, Crouter S, Bassett DR (2004). Pedometer measures of free-living physical activity: Comparison of 13 models. Med Sci Sports Exerc.

[15] Wang Z, Tsujimoto T, Sasai H, Tanaka K (2018). Associations of various exercise types with self-rated health status: A secondary analysis of Sports-Life Data 2012. J Phys Fit Sports Med.

[16] Sasakawa Sports Foundation. SSF Books. https://www.ssf.or.jp/en/publication/ssf_books/index.html. Accessed 27 Dec 2021.

[17] Our world in data. Statistics and research: Coronavirus (COVID-19) cases. https://ourworldindata.org/. Accessed 27 Dec 2021.

[18] Sasakawa Sports Foundation. https://www.ssf.or.jp/en/index.html. Accessed 27 Dec 2021.

[19] Bernal JL, Cummins S, Gasparrini A (2017). Interrupted time series regression for the evaluation of public health interventions: a tutorial. Int J Epidemiol.

[20] Muggeo VMR. Regression models with break-points/change-points estimation; published 2021. https://cran.r-project.org/web/packages/segmented/segmented.pdf. Accessed 21 Dec 2021.

[21] The Tokyo Organising Committee of the Olympic and Paralympic Games. Tokyo 2020 Action & Legacy Report. https://www.2020games.metro.tokyo.lg.jp/special/watching/tokyo2020/games/legacy/. Accessed 8 June 2022

[22] Kamada M, Kitayuguchi J, Abe T, Taguri M, Inoue S, Ishikawa Y (2018). Community-wide intervention and population-level physical activity: a 5-year cluster randomized trial. Int J Epidemiol.

[23] Harris S, Dowling M. Sport participation and Olympic legacies: a comparative study (1st ed.). 2021. Routledge. 10.4324/9781315523774

[24] Brunner E, Cable N, Iso H (2020). Health in Japan: Social epidemiology of Japan since the 1964 Tokyo Olympics.

[25] Aizawa K, Wu J, Inoue Y, Sato M (2018). Long-term impact of the Tokyo 1964 Olympic Games on sport participation: a cohort analysis. Sport Manag Rev.

[26] Global Observatory for Physical Activity. The 2nd Physical Activity Almanac. https://indd.adobe.com/view/cb74644c-ddd9-491b-a262-1c040caad8e3. Accessed 10 Dec 2021.

