# Supplementary material for: Evaluation of pre-Games effects of the Tokyo 2020 Olympic Games on Japanese population-level physical activity: a time-series analysis
Source: Int J Behav Nutr Phys Act. 2022 Aug 6;19:96. doi: 10.1186/s12966-022-01332-x (PMC9356482; doi:10.1186/s12966-022-01332-x)
Supplement: Supplementary file 1 — Additional file 1: Appendix Figure 1. Changes in the trends of population-level physical activity for each survey. NSLS, National Sports Life Survey; NHNS-J, National Health and Nutrition Survey, Japan; POSSP, Public Opinion Survey on Sports Participation of Tokyo residents; EBP, Estimated break-point. Sports participation rate is defined as engaging in sports at least once per week. For assessing exercise habit prevalence, exercise was defined as engaging in exercise activities for ≥30 min/day, ≥2 days/week, over a year. The exercise habit prevalence and average step count data were adjusted for age and gender. When analysing the NSLS data, the residential area was added to the adjustments. The estimated break-points and slope are calculated using segmented regression analysis. a)–c): national sample, d): Tokyo residents. For sports participation rates among Tokyo residents in the NSLS (sub-sample analysis, not shown), there was no estimated break point because there was no change in the linear relationship. [file 12966_2022_1332_MOESM1_ESM.pdf]

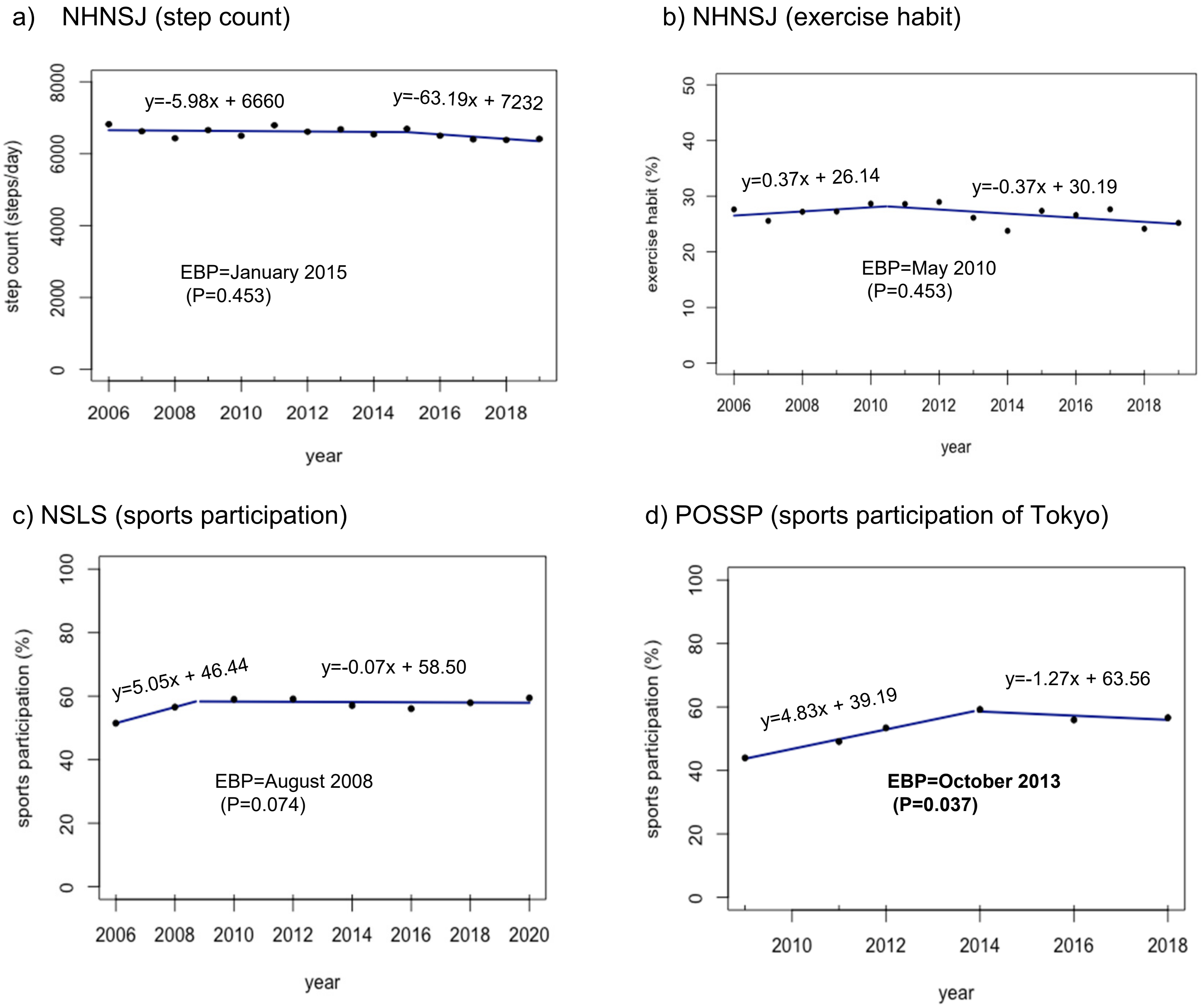

Appendix Figure 1. Changes in trends of population-level physical activity for each survey.
NSLS, National Sports Life Survey; NHNS-J, National Health and Nutrition Survey, Japan; POSSP, Public Opinion Survey on Sports Participation of Tokyo residents; EBP, Estimated break-point. Sports participation rate is defined as engaging in sports at least once per week. For assessing exercise habit prevalence, exercise was defined as engaging in exercise activities for ≥30 min/day, ≥2 days/week, over a year. The exercise habit prevalence and average step count data were adjusted for age and gender. When analysing the NSLS data, the residential area was added to the adjustments. The estimated break-points and slope are calculated using segmented regression analysis. a)–c): national sample, d): Tokyo residents. For sports participation rates among Tokyo residents in the NSLS (sub-sample analysis, not shown), there was no estimated break point because there was no change in the linear relationship.
