# Supplementary material for: Evaluation of pre-Games effects of the Tokyo 2020 Olympic Games on Japanese population-level physical activity: a time-series analysis
Source: Int J Behav Nutr Phys Act. 2022 Aug 6;19:96. doi: 10.1186/s12966-022-01332-x (PMC9356482; doi:10.1186/s12966-022-01332-x)
Supplement: Supplementary file 2 — Additional file 2: Appendix Table 1. Sensitivity analysis for the pre-Games effects of the Tokyo 2020 Olympic Games on sports participation in the National Sports-Life Survey data set. [file 12966_2022_1332_MOESM2_ESM.docx]

**Appendix Table 1.** Sensitivity analysis for the pre-Games effects of the Tokyo 2020 Olympic Games on sports participation in the National Sports-Life Survey data set

|  |  |  | **Pre (2008–2012)** | |  | **Post (2013–2018/2020)** | |  | **Change** | |
| --- | --- | --- | --- | --- | --- | --- | --- | --- | --- | --- |
|  | **Survey year** | **Data** | **Mean*** | **(95% CI)** |  | **Mean*** | **(95% CI)** |  | **Mean*** | **(95% CI)** |
| **Sports participation (%)** |  |  |  |  |  |  |  |  |  |  |
| Overall | **2008–2018** | A | 58.7 | (57.2; 60.2) |  | 56.5 | (55.0; 58.0) |  | -2.2 | (-12.4; 8.0) |
| Male | **2008–2018** | A | 57.4 | (56.9; 57.9) |  | 56.0 | (55.5; 56.5) |  | -1.4 | (-3.3; 0.4) |
| Female | **2008–2018** | A | 59.5 | (57.7; 61.3) |  | 57.4 | (55.6; 59.3) |  | -2.1 | (-8.5; 4.3) |
| **Sports participation (%)** |  |  |  |  |  |  |  |  |  |  |
| Overall | **2008–2018** | I | 58.1 | (56.9; 59.3) |  | 56.9 | (55.5; 58.4) |  | -1.2 | (-2.8; 0.4) |
| Male | **2008–2018** | I | 57.2 | (56.2; 58.2) |  | 56.1 | (55.0; 57.1) |  | **-1.1** | **(-1.9; -0.3)** |
| Female | **2008–2018** | I | 59.0 | (57.0; 61.1) |  | 57.8 | (55.8; 59.7) |  | -1.3 | (-3.9; 1.3) |
| **Sports participation (%) in Tokyo (sub-sample)** |  |  |  |  |  |  |  |  |  |  |
| Overall | **2008–2018** | I | 61.9 | (57.7; 66.2) |  | 59.6 | (58.8; 60.3) |  | -2.3 | (-6.6; 1.9) |
| Male | **2008–2018** | I | 60.6 | (55.5; 65.7) |  | 55.5 | (53.7; 57.3) |  | -5.1 | (-10.5; 0.2) |
| Female | **2008–2018** | I | 63.2 | (58.7; 67.7) |  | 63.7 | (60.6; 66.8) |  | 0.5 | (-4.9; 5.9) |
| **Sports participation (%)** |  |  |  |  |  |  |  |  |  |  |
| Overall | 2008–2020 | A | 59.2 | (57.0; 61.5) |  | 56.8 | (55.1; 58.6) |  | -2.4 | (-11.4; 6.6) |
| Male | 2008–2020 | A | 57.6 | (56.7; 58.6) |  | 56.4 | (55.6; 57.2) |  | -1.2 | (-3.7; 1.2) |
| Female | 2008–2020 | A | 59.8 | (57.8; 61.9) |  | 58.0 | (56.3; 59.7) |  | -1.8 | (-7.2; 3.6) |
| **Sports participation (%)** |  |  |  |  |  |  |  |  |  |  |
| Overall | 2008–2020 | I | 58.1 | (56.9; 59.3) |  | 57.5 | (56.1; 58.9) |  | -0.6 | (-2.4; 1.2) |
| Male | 2008–2020 | I | 57.1 | (56.2; 58.0) |  | 56.5 | (55.4; 57.6) |  | -0.6 | (-1.7; 0.6) |
| Female | 2008–2020 | I | 59.1 | (57.0; 61.2) |  | 58.5 | (56.6; 60.5) |  | -0.6 | (-3.3; 2.1) |
| **Sports participation (%) in Tokyo (sub-sample)** |  |  |  |  |  |  |  |  |  |  |
| Overall | 2008–2020 | I | 61.9 | (57.6; 66.2) |  | 59.3 | (58.5; 60.1) |  | -2.6 | (-6.9; 1.7) |
| Male | 2008–2020 | I | 60.6 | (55.5; 65.7) |  | 54.9 | (53.1; 56.6) |  | **-5.7** | **(-11.1; -0.4)** |
| Female | 2008–2020 | I | 63.2 | (58.7; 67.7) |  | 63.7 | (61.5; 66.0) |  | 0.5 | (-4.5; 5.6) |

CI, confidence interval; A, aggregated data; I, individual data. *Mean values and regression models were adjusted for age, gender, and residential area. Bold indicates P < 0.05.
